# Automated thermal gradient test for unprovoked assessment of nociceptive preference in rodents

**DOI:** 10.3389/fnbeh.2025.1709160

**Published:** 2025-12-05

**Authors:** Thomas Deakin, Shoupeng Wei, Yao Wang, Raina E. Rhoades, Tommy S. Tillman, Pei Tang, Yan Xu

**Affiliations:** 1 Department of Anesthesiology and Perioperative Medicine, University of Pittsburgh, Pittsburgh, PA, United States; 2 Department of Pharmacology and Chemical Biology, University of Pittsburgh, Pittsburgh, PA, United States; 3 Department of Structural Biology, University of Pittsburgh, Pittsburgh, PA, United States; 4 Department of Physics and Astronomy, University of Pittsburgh, Pittsburgh, PA, United States

**Keywords:** self-reported pain behaviors, automated pain detection, thermal gradient, thermal preference, pain assessment

## Abstract

In animal models, reflexive responses to noxious stimuli (e.g., paw withdrawal in von Frey, Hargreaves, or cold plantar tests) are largely spinal reflexes and their quantitative measures (latency or threshold) may not directly reflect clinically relevant pain perception as assessed by human quantitative sensory testing, which captures both conscious sensory and affective components of pain as a subjective experience. This study aims to develop a complementary behavioral testing strategy for rapidly and automatically detecting rodents’ thermal responses under different pain conditions without human interference. A device is engineered to create a linear thermal gradient from 4 °C to 58 °C along a long aluminum floor of four equal-size corridors, each having a dimension of 137 cm × 10 cm × 22 cm (*L* × *W* × *H*) and allowing four freely roaming rodents to be simultaneously evaluated to increase the throughput of *in vivo* pain testing. Animal behaviors influenced by the temperature gradient are recorded by a camera and analyzed using ANY-Maze. The duration of data collection is investigated, showing that the data collected in as short as 10 min can adequately capture thermal preferences of mice along the temperature gradient. Animal behaviors reveal differences in thermal nociception between male and female mice, capture counterintuitive changes in nociceptive thermal avoidance in the absence and presence of inflammatory pain, and show analgesic effects of morphine (10 mg/kg subcutaneously) as well as its stimulation of hyperactive locomotion. The sensitivity, reliability, and efficiency of the new thermal gradient test will not only help mechanistic investigations of various thermal sensing receptors but also enable high-throughput *in vivo* pain evaluation and analgesic drug screening for developing new treatments for pain management.

## Introduction

Clinical pain management remains a formidable challenge. An estimated 21% of the population in the US is living with chronic pain (Rikard et al., 2023). Currently used pain medications often have unwanted side effects and misuse potential that partially contributes to the opioid epidemic (Kolodny et al., 2015). Preclinical pain research plays a significant role in understanding the mechanisms underlying various pain conditions as well as possible treatment strategies for pain management. Sensitive and objective behavior tests in preclinical animal models are essential for accurate pain assessment and for the discovery and development of effective non-opioid analgesics (Gonzalez-Cano et al., 2020; Sadler et al., 2022; Zhang et al., 2022).

Peripheral thermal sensation enables mammals to perceive temperatures and react to noxiously hot or cold stimuli (Vriens et al., 2014). The thresholds to heat and cold tolerance can vary under different physiological and pathological conditions (Wang et al., 2014). Because of this, thermal sensitivity assessments are routinely used in preclinical pain research. The Hargreaves test, for example, is commonly used to assess thermal pain sensation in rodents (Hargreaves et al., 1988). While useful, the Hargreaves test has many limitations (Deuis et al., 2017; Sadler et al., 2022). As pointed out by Deuis et al. (2017), a major challenge in interpreting behavioral nociceptive assays lies in distinguishing spinally mediated reflex responses from true pain perception, which involves higher-order neural processing. Reflexive behaviors, such as paw or tail withdrawal, primarily reflect activation of segmental spinal circuits responsible for protective motor outputs. In contrast, pain perception requires ascending transmission through the spinothalamic and spinoparabrachial pathways to supraspinal regions, including the thalamus, somatosensory cortex, and limbic structures. Importantly, the anatomical site of stimulation determines which neural circuits are engaged. For example, hind paw testing using von Frey or Hargreaves assays primarily activates lumbar spinal segments and relatively simple reflex arcs. Consequently, differences in withdrawal thresholds or latencies across body regions may not indicate true variations in “pain sensitivity” but rather reflect differences in local innervation density, fiber types, and spinal connectivity. Therefore, quantitative outcomes from reflex-based tests should be interpreted within the anatomical and neurophysiological context of the stimulated site. Integrating these data with assays that assess affective-motivational components of pain, such as conditioned place aversion or operant tasks, offers a more comprehensive evaluation of pain-relevant processing across multiple levels of the nervous system (Deuis et al., 2017; Mogil, 2022).

Technically, administering the Hargreaves test is labor intensive and data quality relies heavily on the experience of individuals performing the test to differentiate whether a withdrawal is due to pain or merely a supraspinal reflex (Rice et al., 2008). Human intervention and confined spaces needed for the Hargreaves test frequently trigger anxiety in rodents that may also compromise accuracy of pain assessments (Deuis et al., 2017).

Compared to the Hargreaves test, the thermal place preference test (TPPT) offers significant progress in automated and noninvasive assessment of thermal sensitivities in rodents under different pain conditions (Moqrich et al., 2005; Balayssac et al., 2014; Caporoso et al., 2020). In TPPT, rodents move freely between two or more visually indistinguishable compartments of different surface temperatures. Human intervention is reduced to a minimum so that animals experience less stress and anxiety. Thus, TPPT is more likely to offer an unbiased assessment of thermal sensitivity and hypersensitivity under different pain conditions and has a potential application in high-throughput *in vivo* drug screening (Caporoso et al., 2020). Despite these improvements, pairing temperatures for different compartments in TPPT is often nontrivial and time consuming due to activation of different direct or indirect thermal sensing receptors, including the transient receptor potential (TRP) channel subclasses (TRPA1, TRPM8, TRPV4, TRPV3, TRPV1, and TRPV2 for different temperatures from 0 °C to >52 °C) (Qi et al., 2025). It often requires a rigorous searching process to optimize temperature pairs in TPPT for different pain models.

To further develop methods for automated evaluation of thermal sensitivity in rodents, we developed a thermal gradient test (TGT) in the current study. TGT offers unparallel advantages by eliminating the requirement for choosing temperature pairs. New experimental parameters and pain dimensions can also be explored to differentiate time-aggregated pain experience from nociception, hence providing a more clinically relevant pain assessment than traditional reflex-based measures. Here, we report a new design of TGT that not only enables automated detection of thermal sensitivities under different pain conditions but also provides unbiased results simultaneously for multiple rodents. The reliability, sensitivity, and efficiency of this new TGT assay are desirable for enhancing preclinical pain research and more importantly, for making the rapid *in vivo* screening of novel analgesics feasible.

## Materials

### Mice

All procedures were approved by the Institutional Animal Care and Use Committee of the University of Pittsburgh (D16-00118). C57BL/6J mice, aged 12 weeks or 30 weeks, were purchased from Jackson Laboratories. The mice were habituated for two days upon arrival before being used for experiments. They were housed in a temperature-controlled (22 ± 1 °C) and humidity-controlled (50 ± 10%) room under a 12-h light/dark cycle (light on at 7:00 a.m.). Water and food were provided *ad libitum*. Animals with clear health concerns, such as fighting wounds, were excluded due to potential impact on pain sensitization. A total of 30 male and 47 female mice were included in this project, but one female was excluded at a later stage due to fighting wounds. Given the clinical and translational relevance to inflammatory pain in the aging populations (Norton et al., 2024), particularly in women (Wranker et al., 2016; Puto et al., 2024), we used 11 middle-aged (30 weeks old) female mice for the complete Freund’s adjuvant (CFA)-induced inflammation experiment. Prior to CFA injection, these mice underwent the TGT to establish baseline data, which were analyzed using the same methods applied to post-injection measurements.

### Complete Freund’s adjuvant for inflammatory pain model

CFA was purchased from InvivoGen (vac-cfa-10). Each mouse in the CFA group was injected with 25 μL of CFA to the left hind paw to develop inflammatory pain. For CFA injection, mice were anesthetized within 60 s with 5% isoflurane (1 L/min). After CFA injection, mice were kept outside their home cage for 2–5 min on a 37 °C warming pad until fully recovered from anesthesia. The CFA-injected mice were tested 24 h after injection.

### Morphine treatment

Injectable morphine sulfate (10 mg/mL) was purchased from Covetrus North America (cat# 057202). For a more controllable injection volume, the original morphine sulfate was diluted to 5 mg/mL using sterile phosphate-buffered saline (PBS, pH = 7.4, Sigma Aldrich P3813-10PAK). Mice were restrained during subcutaneous morphine administration. All behavior tests were performed 30 min after the morphine injection. For repeated measurements on the same mice, a minimum 48-h waiting period was given to ensure complete morphine clearance.

## Methods

### Data collection and analysis

ANY-Maze records videos of TGT experiments at 15 frames per second. Typically, we exclude the first 30 s of the video from the data analysis, considering the heavily weighted exploratory behavior of mice during that period. Thus, a 10-min TGT recording for each mouse produces 8,550 data points that are analyzed by ANY-Maze for distance traveled and time spent in different zones along the temperature gradient. Prism 9.4.0 software (GraphPad) was used for statistical analysis and for data presentation in all figures. Significance was determined by the two-tail heteroscedastic or paired *t* test and the extra sum of squares *F* test depending on the chosen testing parameters, as detailed in the main text and figure legends. A value of *p* < 0.05 is considered significant. To estimate the number of animals needed for different comparisons, we evaluated anticipated effect size relative to the within-group standard deviation *σ* using Cohen’s *d* calculations (Cohen, 1992): *d* = Δ*μ*/*σ*, where Δ*μ* represents the detectable mean difference of a given variable between groups, and σ is the pooled standard deviation of the population calculated by the equation $\sigma =\sqrt{\frac{\left(\mathrm{SS}\_\text{male}+\mathrm{SS}\_\text{female}\right)}{\left(\mathrm{DFd}\_\text{male}+\mathrm{DFd}\_\text{female}\right)}}$ , with SS and DFd indicating the sum of squares and the degrees of freedom, respectively. To detect the sex difference, we anticipate a ~1.5 °C difference in the temperature preference based on pilot studies when the pooled σ within group is ~1.2 °C. Cohen’s *d* estimates *N* ≥ 11/group to achieve 80% power with *α* = 0.05. Similarly, for CFA inflammation evaluation, a large effect size of >2 °C is expected, and Cohen’s *d* gives *N* = 6/group to achieve 80% power with *α* = 0.05. However, using pooled *σ* from nonlinear/mixed models is known to potentially lead to an inflated *d* and consequently overestimate expected power (Hoenig and Heisey, 2001; Lazic et al., 2018) because the actual between-subject variance is not entered into the denominator. Thus, we increased N as reported in this manuscript to achieve a robust and reliable comparison between groups.

### TGT apparatus design and fabrication

We engineered an improved and easily scalable thermal gradient device with four equal-size chambers (length, 137 cm; width, 10 cm; and wall height, 22 cm) atop an aluminum plate (length, 157 cm; width, 45 cm; and thickness, 0.95 cm) with a stable temperature gradient along the length of the plate. The top surface of the aluminum plate, smoothed by 3-micro polishing films (Thomas Scientific, NJ, United States), serves as the floor of the chambers. The bottom surface of the plate rests on a 3.8-cm thick thermal insulating foam to maintain temperature stability over time. The walls of the chambers are made of black plastic boards with a matte finish and no reflection. Four 144-W core semiconductor thermoelectric Peltier units (Walfront Electronics, Wuhan, China) are press-mounted to the top surface at each end of the corridors. Two DC power supplies (B&K Precision, Model 1900B, 0–16 VDC and 0–60 A, Yorba Linda, CA) separately regulate Peltier devices at the two ends of the plate and provide ~14 V (36.8 A) and ~6.3 V (12.8 A) to create a linear thermal gradient ranging from 4 °C to 58 °C across the long axis, as confirmed by surface temperature measurements using a thermocouple (Grainger, Model GK11M, 3LRX4) that is connected to a Temperature Monitor (Physitemp Instruments Inc., Thermalert Model TH-8, Clifton, NJ). Photos of the TGT device are shown in Supplementary Figure S1.

Mice are always placed in the middle of the gradient at the beginning of the TGT and allowed to roam freely along the corridors. The center-of-mass body position and accumulative time spent by individual mice in each temperature zone, determined experimentally as detailed below in the Results section, are tracked by a video camera mounted above the TGT device and recorded using ANY-Maze (Stoelting Co., Wood Dale, IL, United States). The automated nature of the data collection and analyses makes blinding unnecessary.

### The TGT assay

All mice were habituated and allowed to move freely within individual chambers at room temperature (20 ± 1 °C) for >10 min one or more days before TGT evaluations. The average body length of a mouse is approximately 8 cm without considering the tail. However, during the TGT, mice are actively running rather than remaining stationary. This movement causes their body length to appear longer due to front and hind paws touching a wider temperature range when the center of mass is evaluated from the video recordings. Therefore, we used the effective body length of a moving mouse as the basis for dividing the full length of the corridor into 14 zones, each measuring 9.8 cm. The percent time distribution in the 14 zones shows a U-shaped curve along the long axis of the chambers (Supplementary Figure S2), reflecting the natural tendency of mice to prefer the corners at the two ends.

**Figure 1:**
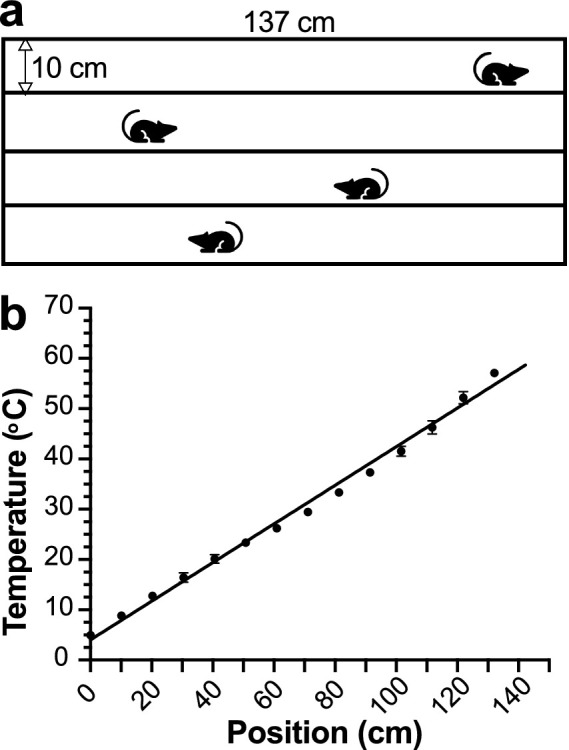
The thermal gradient test (TGT) apparatus. **(a)** Schematic of the TGT device with four measurement corridors. **(b)** A linear relationship (*Y* = 0.38*X* + 4.03, *R*^2^ = 0.99) between distance (0–137 cm) and temperature (4 °C–58 °C), determined for individual corridors through independent measurements by three investigators.

The device allows for simultaneous TGT measurements for four mice (Figure 1A). The gradient in each corridor was measured in individual zones using a calibrated thermocouple during the initial temperature calibration and verified before and after each test to ensure a stable and reproducible linear thermal gradient. The uniform thermal conductivity of the plate ensures that the local surface temperature at a given position along the gradient does not deviate appreciably from the extrapolated setpoint, thereby providing a spatially stable and linear thermal profile across testing zones (Figure 1B). Linear least squares fit of temperatures as a function of the center position of each zone along the long axis of the corridor (Figure 1B) converts the animals’ center-of-mass body positions to the averaged zone temperatures being experienced. We also perform calibration to adjust for any minor and unexpected fluctuations in the room temperature.

**Figure 2:**
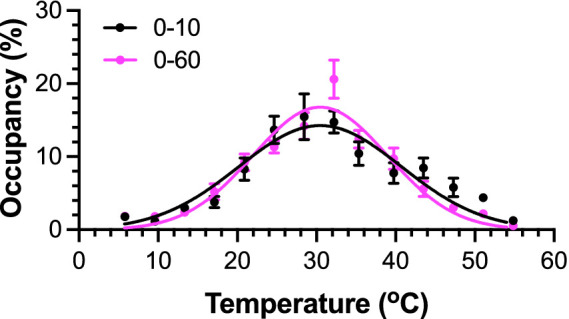
Mice show temperature preference within 10 min of TGT. The Gaussian curve fittings of the first 10-min or the entire 60-min data resulted in *T*_peak_ values with no statistical differences (*p* = 0.997). Data were obtained from naïve C57BL/6J male mice (*n* = 12) and are reported as mean ± SEM. At extreme temperatures, the SEM is smaller than the symbol size.

The measurement duration in traditional thermal preference tests varied widely in previously published investigations, ranging from a few minutes to several hours (Lee et al., 2005; Moqrich et al., 2005; Caporoso et al., 2020). To achieve both accuracy and efficiency for *in vivo* screening of new analgesics, we analyzed TGT data by sliding time windows of data collection in a subset of 12 male mice, which were subjected to 60 min of data collection. A Gaussian distribution of temperature preference emerges in less than 10 min, showing rodents’ avoidance of cold (<18 °C) or hot (>42 °C) zones at two ends of the corridors (Figure 2) and countering their natural behavioral tendency toward the corners. Two-parameter Gaussian fitting results in the most preferred temperature (*T*_peak_), at which the mice spend most time during data collection. The data from the first 10 min and the full 60 min show *T*_peak_ values of 30.5 ± 0.7 °C and 30.5 ± 0.4 °C, respectively (Figure 2), which are not statistically different [extra sum of squares *F* test, *F*(1, 330) = 1.38 × 10^−5^; *p* = 0.997]. The fitting also reports the width of the Gaussian distribution, or the standard deviation (SD) of the distribution. As shown in Figure 2, the SD decreases when the data collection time extends, changing from 10.3 ± 0.7 °C for 10 min to 8.5 ± 0.4 °C for 60 min. This change is statistically significant [*F*(1, 330) = 4.424; *p* = 0.0362]. Since the total area under the curve is 100%, a narrower distribution corresponds to a higher peak zone occupancy. Thus, the percent peak occupancy is a dependent variable of SD, changing slightly from 14.3 ± 0.8% for 10 min to 16.8 ± 0.7% for 60 min (Figure 2). These changes are primarily due to rodents’ learning of their preferred temperature zones over a long testing time and subsequently spending more time or “resting” with extended immobilization at their preferred temperature zone to avoid the cold or hot zones at the two ends of corridors during the later period of the 60-min data collection. Indeed, we observed a significant increase in the percentage of accumulative resting time (>3 s) with increasing test duration (54.0 ± 3.7% for 60 min versus 43.6 ± 2.8% for the first 10 min, *p* = 0.036, Supplementary Figure S3). The distribution of >3 s resting times as a function of zones follows a similar distribution to the accumulative occupancy times in different zones (Supplementary Figure S4A), suggesting that extending the measurement time beyond 10 min only reaffirms the animals’ choice of the preferred temperatures.

## Results

### TGT detects sex differences in thermal sensitivity and temperature preference in mice

**Figure 3:**
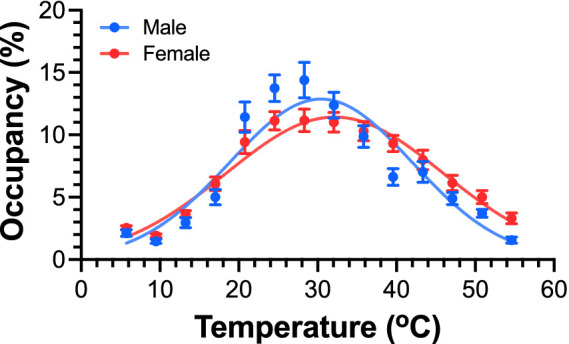
Thermal sensitivity and preference of male and female mice revealed by TGT. Occupancy of mice in different zones along the temperature gradient in a 10-min assay exhibits a Gaussian distribution. Naïve male (*n* = 30, blue) and female (*n* = 36, red) C57BL/6J mice show significantly different *T*_peak_ values (male 30.4 ± 0.3 °C vs. female 32.2 ± 0.4 °C) according to an extra sum of squares *F* test [*F*(1, 895) = 19.50; *p* < 0.0001]. Data are presented as mean ± SEM.

Males and females perceive pain differently according to previous human and animal studies (Kaikaew et al., 2017; Boerner et al., 2018; Smith, 2019). Here, we use TGT to evaluate thermal preference of naïve male and female mice to validate the sensitivity of the TGT method. Gaussian fitting of the TGT data from these two groups of mice (Figure 3) resulted in significantly different *T*_peak_ values with 30.4 ± 0.3 °C for male and 32.2 ± 0.4 °C for female mice [*F*(1, 895) = 19.50; *p* < 0.0001]. The corresponding Gaussian distribution widths are also statistically different between males (SD = 11.6 ± 0.4 °C) and females (SD = 13.8 ± 0.4 °C) [*F*(1, 895) = 22.27, *p* < 0.0001]. This is also reflected in the maximum occupancies of 12.9 ± 0.8% and 11.4 ± 0.7% for males and females, respectively. The power analysis shows Cohen’s *d* of 1.23, suggesting a large effect size.

### TGT captures inflammatory pain induced by complete Freund’s adjuvant

**Figure 4:**
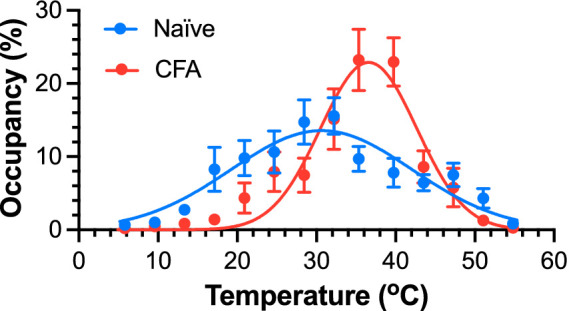
TGT captures a distinctly different thermal response from mice with and without CFA inflammation of the hind paw. Significant differences in *T*_peak_ [32.5 ± 0.8 °C vs. 36.7 ± 0.5 °C; *F*(1, 302) = 8.14; *p* < 0.0046] and Gaussian SD [14.3 ± 0.9 °C vs. 6.1 ± 0.5 °C; *F*(1, 302) = 46.09; *p* < 0.0001] were observed in female C57BL/6J mice without CFA (naïve, blue) and 24-h after CFA injections (CFA, red). Data are reported as mean ± SEM (*n* = 11). At extreme temperatures, the SEM is smaller than the symbol size.

Changes in thermal sensitivity, including cold allodynia and heat hypersensitivity, are often related to pain conditions. To test whether the TGT assay can quantitatively capture pain states in mice, we compared the results from female mice 24-h after intraplantar CFA injection in the left hind paw against the female naïve controls. We observed drastically different Gaussian distributions (Figure 4) with a Cohen’s *d* of 1.73 for a large effect size. *T*_peak_ shifted significantly from 32.5 ± 0.8 °C (naïve) to 36.6 ± 0.5 °C (CFA) [*F*(1, 302) = 8.14; *p* < 0.0046], and SD is significantly narrowed from 14.3 ± 0.9 °C for the naïve controls to 6.1 ± 0.5 °C for the CFA mice [*F*(1, 302) = 46.09; *p* < 0.0001]. With narrowing of the distribution, the corresponding peak occupancy of the comfortable zone increased from 11.2 ± 0.5% to 22.9 ± 1.7% of the measurement time. The narrower distribution in the CFA group than in the naïve controls results primarily from a significantly lower occupancy in zones with temperatures <30 °C and >45 °C (Figure 4) and significant increase of immobile time of the CFA mice in the “comfortable” temperature zones (Supplementary Figure S4B). This observation is consistent with reported changes in thermal sensitivities of mice following CFA inflammation of the hind paw (Allchorne et al., 2005). The CFA-induced peripheral inflammation is known to elicit cold (Allchorne et al., 2005) and warm allodynia and heat hyperalgesia (Hargreaves et al., 1988; Dirig et al., 1997). In addition to heightened aversive sensitivity to thermal extremes due to CFA-induced inflammation, reduced spontaneous locomotor activity and exploratory behavior may secondarily decrease occupancy in the extreme hot or cold zones. Therefore, the diminished presence in noxious temperature areas likely reflects a combined effect of altered nociception and suppressed locomotion.

### TGT reveals complex behavioral effects of morphine

**Figure 5:**
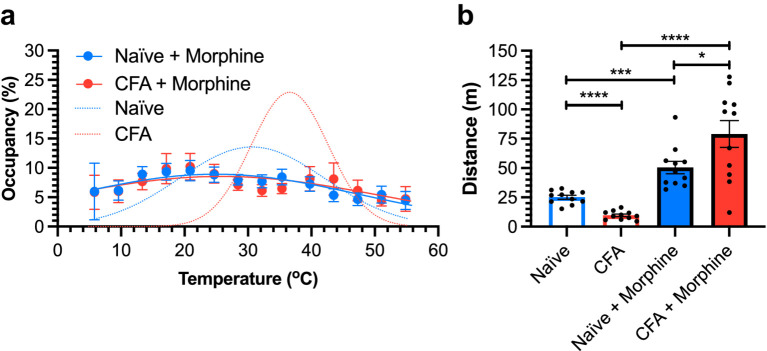
Morphine effects revealed by TGT. **(a)** Temperature zone occupancy distributions are plotted for female naïve + morphine (blue) and CFA + morphine (red) C57BL/6J mice 30-min post morphine injection (10 mg/kg, s.c.). The occupancy distributions of morphine-free mice from Figure 4 are included for comparison (dotted lines, blue: naïve, red: CFA). **(b)** Morphine induces hyperactive locomotion in both naïve + morphine (blue) and CFA + morphine (red) mice, which traveled significantly greater distances than their morphine-free counterparts according to the two-tailed unpaired (naïve) or paired (CFA) *t*-tests. Additionally, CFA + morphine mice traveled a significantly greater distance than naïve + morphine mice. All data are presented as means ± SEM (*n* = 11). ^*^p < 0.05, ^***^p < 0.001, ^****^p < 0.0001, and ^ns^*p* > 0.05.

Morphine is widely used as an analgesic in humans and in animal research. The adverse effects associated with morphine are recognized (Kim et al., 1998; Glare et al., 2006; Morgan et al., 2012; Caporoso et al., 2020). Here, we use TGT to evaluate effects of morphine (10 mg/kg, subcutaneously) on mice under different pain conditions, either pain free (naïve mice) or experiencing CFA-elicited inflammatory pain. TGT was performed 30 min after morphine injection. In stark contrast to the narrow Gaussian distributions of the CFA mice before morphine injection (Figure 4), the CFA mice with morphine treatment presented an almost flat distribution with a roughly equal occupancy along the temperature gradient (Figure 5A). The nearly flat distribution was also observed in naïve mice after the morphine injection. The occupancy at the cold and hot ends of the temperature gradient increased from complete avoidance (0%) to nearly equal to other temperature zones (4–6% compared to the theoretical equal distribution of 7.1% in 14 zones) when mice were under the influence of morphine (Figure 5A), suggesting that morphine significantly diminishes not only thermal hyperalgesia in mice experiencing CFA-induced inflammatory pain, but also the temperature sensitivity of the naïve mice (Figure 5A).

In addition to the morphine-induced inhibition of thermal hyperalgesia or hypersensitivity, TGT also revealed morphine-induced hyperactive locomotion in mice. Both the naïve + morphine and CFA + morphine mice exhibited increased locomotor activity, which was quantified by the distances traveled within a defined time window (e.g., 10 min) (Figure 5B). The observation is consistent with previous reports that morphine can induce hyperactivity in rodents (Caporoso et al., 2020; Acevedo-Canabal et al., 2023).

## Discussion

In this study, we demonstrated a streamlined protocol to evaluate nociceptive thermal avoidance in rodents by unprovoked and self-reported behaviors of temperature preference in an automated fashion. The results suggest that a 10-min TGT can capture essential information (e.g., *T*_peak_) comparable to that revealed in significantly longer durations (e.g., 60 min), thereby enabling a significantly higher throughput of *in vivo* behavioral pain evaluation than the conventional methods on non-communicating animals. As shown in Figure 2, a TGT with 10-min data collection is adequate for determining *T*_peak_, which is often all that is needed for *in vivo* screening of new analgesics in a pain model. However, optimal durations for TGT data collection depend on emphases of different applications. For drug screening, a reasonable balance between accuracy and efficiency dictates the short duration so that many compounds and animals can be screened quickly and cost-effectively.

There are two major commercial devices for automated detection of thermal sensitivity in rodents. One is the thermal gradient ring (TGR), which is designed using Zimmermann’s method (Touska et al., 2016) and sold by Ugo Basile, Animalab, and ANY-maze. TGR removes potential biases from edge-seeking behavior that may occur in linear assays. However, as shown in our data, edge-seeking is not an issue in detection with a linear thermal gradient. Mice spent a minimal time at both ends of the plate to avoid cold and hot temperatures. Additionally, our TGT design offers several advantages. *First*, our TGT device covers 4 °C–58 °C continuously. All commercially available TGRs require the experimenter to pre-define the temperature range before the trial begins, either from room temperature to 65 °C using the heating device or from 4 °C to 35 °C using the heating/cooling device. *Second*, a commercial TGR allows only one rodent at a time in each trial, but ours can test four rodents simultaneously, significantly increasing efficiency that is crucial for screening new analgesic candidates *in vivo*. *Third*, the cost for our TGT device is only a fraction of the price for a commercial TGR system, making this cost-effective method more broadly accessible to the pain research community. The second and third advantages of our TGT system are also true when compared to a commercial TGT system (HBIO-Panlab). Furthermore, our TGT device has a much longer dimension (0–137 cm) and broader temperature range (4–58 °C), providing a significantly better temperature resolution [(58 − 4)°C/137 cm = 0.39 °C/cm] than the commercial device. Thus, our TGT system is a welcome addition to pre-clinical pain research considering the temperature range and resolution, efficiency in data turnover, and cost-effectiveness. Supplementary Table 1 presents a comparison between our TGT and traditional assays, including the Hargreaves and hot plate tests.

Female rodents are known to prefer slightly warmer temperatures than males (Gaskill et al., 2009). We used this known sex difference on temperature preference to gauge the measurement sensitivity of TGT. The observed preference of female mice to a higher *T*_peak_ is consistent with a previous report using a different experimental method (Kaikaew et al., 2017), in which mice were allowed to choose between two temperatures (29 °C vs. 32 °C) and showed that female mice resided much longer at 32 °C than at 29 °C and behaved significantly differently from their male counterparts in the same experimental setting (Kaikaew et al., 2017). Note that a large number of female mice (*n* = 36) was used to mitigate the potential influence of estrous cycle variability on sex-dependent responses, as estrous stages were not closely monitored. Our TGT data not only confirm the different *T*_peak_ between male and female mice but also reveal an overall narrower tolerable temperature range and stronger preference of 22–30 °C for male mice. The underlying mechanism for this sex difference remains unknown and warrants further investigation, considering that the difference in thermal preference between males and females has been supported and validated by different experimental methods and independent research laboratories.

The results in Figure 4 comparing the animal behaviors with and without CFA-induced inflammatory pain illustrate added dimensions of TGT in pain assessments that are difficult to obtain with the conventional pain evaluation methods. Traditional thermal sensitivity tests, such as Hargreaves and acetone tests, can detect allodynia or hypersensitivity after inflammation based on shortened latencies of supraspinal reflexes as a nociceptive nerve process but cannot report thermally induced pain as an experience when animals are given a wide range of temperature options. The shift of preferred temperature (*T*_peak_) to nearly the body temperature with a significantly narrower distribution under CFA-induced inflammation is unexpected based on the perceived hypersensitivity to heat under inflammation. However, our observation is consistent mechanistically with the known interplay among different subtypes of TRP channels that are responsible for temperature sensing. Extensive investigations have been carried out to understand thermo-TRP channels for sensing various temperatures (Qi et al., 2025), showing that TRPA1 (ankyrin 1), TRPM8 (melastatin 8), TRPV4 (vanilloid 4), TRPV3, TRPV1, and TRPV2 are responsible for cold and heat sensitivity at temperatures <17 °C, 15–28 °C, 25–35 °C, 32–39 °C, >42 °C, and >52 °C, respectively. Under the normal physiological conditions, the preferred temperature range for female mice (~32 °C) is primarily regulated predominantly by TRPM8, TRPV4, and TRPV3. While there is evidence that the expression levels of TRPV3 in keratinocytes and TRPV4 in dorsal root ganglia are upregulated after CFA-induced inflammation (Chen et al., 2012; Gazerani et al., 2016), their involvement in warm allodynia remains controversial (Huang et al., 2011). Knockout (KO) studies showed that thermal sensitivity is similar between the wildtype and TRPV3/TRPV4 KO mice after CFA-induced inflammation, suggesting that TRPV3 and TRPV4 have limited contributions to warm allodynia (Huang et al., 2011). The interactions among other thermo-TRP channels, particularly TRPM8, TRPA1, and TRPV1, have been suggested recently as a potential cause of cold and warm hypersensitivity after inflammation (Yang et al., 2023). Using the same TGT concept with a different device design, Li et al. (2021) found that TrpV1-ChR2 mice preferred ~2.3 °C warmer temperature after suprathreshold stimulation, which increased firing of TrpV1^+^ afferents and initiated central sensitization. This “unexpected” result is consistent with our finding in CFA mice (Figure 4) and supports the involvement of TrpV1 in CFA-induced changes in thermal sensitivity. A shift toward warmer temperatures may reflect a coping or analgesic-seeking response that counterbalances sensitized C-fibers by mild external warmth. Innocuous warmth can desensitize TrpV1 (Joseph et al., 2013; Luo et al., 2019) and engage spinal circuits that suppress nociception via activation of inhibitory tones (Kim et al., 2012; Wang et al., 2022). Warmth also suppresses and desensitizes TrpA1 that can attenuate persistent pain at the site of injury (Wang et al., 2012). The precise mechanisms driving the shift toward warmer temperatures detected by the automated self-reporting paradigm await further investigation.

It is worth noting that TGT can detect not only analgesic action of a drug but also other central nervous system effects that go beyond thermal nociception. As shown in Figure 5B, before morphine treatment, the CFA mice traveled significantly shorter distances than the naïve mice due to inflammatory pain (9.7 ± 1.1 m vs. 25.1 ± 1.7 m, *p* < 0.001). Interestingly, after morphine injection, the CFA + morphine mice traveled significantly longer distances than the naïve + morphine mice (79.0 ± 11.5 m vs. 50.4 ± 5.4 m, *p* = 0.0273). The opioid-induced increase in locomotor activity is often attributed to increased dopamine neurotransmission within the mesolimbic system (Broekkamp et al., 1979; Kelley et al., 1980; Kalivas et al., 1983; Hnasko et al., 2005; Botz-Zapp et al., 2021). Why did the CFA + morphine mice show significantly higher locomotor activity than the naïve + morphine mice? Pain sensitization, such as in CFA-induced inflammatory pain, can disinhibit the mesolimbic dopamine circuit (Taylor et al., 2016; Ma et al., 2023) and alter the population and function of dopamine receptors in the striatum (Kienast et al., 2008; Wawrzczak-Bargiela et al., 2020; Simpson et al., 2022), albeit the pain-related changes in dopamine receptors are complex and content-dependent. The nigrostriatal dopamine pathway (Bjorklund and Stenevi, 1979; Haber et al., 2000) is likely responsible for the elevated morphine effect on locomotion of mice experiencing inflammatory pain (CFA + morphine), but this mechanism requires a more in-depth investigation to elucidate specific pathways. Similarly, since response to opioid treatment is often used as a surrogate measure of nociception and pain (Fatt et al., 2024), it would be interesting in future studies to investigate if CFA-induced pain in other areas of the body not in direct contact with the thermal gradient surface, such as in the orofacial region, can trigger altered thermal avoidance behaviors due to central sensitization. The unprovoked behaviors in TGT enable such mechanistic investigations that are impossible by the traditional thermal sensitivity tests based on provoked reflexes. Given that morphine administration in our experiments altered both thermal preference and locomotor activity (Figure 5), future studies incorporating an analgesic that does not affect locomotor activity would further strengthen the specificity of TGT in assessing thermal nociception and pain.

It should also be noted that TGT captures animals’ self-reported thermal preference, which is likely reflecting the comfortable temperature range experienced by the animals under normal and pathological conditions. Clinically, distinctions between discomfort and pain have been made (Ashkenazy and DeKeyser Ganz, 2019), where not all thermal discomforts are associated with pain. Hence, changes in *T*_peak_ can potentially be influenced by the ambient temperature to account for animals’ seeking for “more comfortable” temperatures relative to the “normal” steady-state environment. For this reason, all animals in the current study were housed with room temperatures closely monitored and regulated. Thermal avoidance, as revealed by the changes in the SD of the Gaussian distribution, may depend more directly on nociception and pain experience.

## Conclusion

We present a newly designed, automated thermal gradient test for high-throughput *in vivo* assessments of thermal nociception and pain in rodents without human interference. The new method can differentiate nociceptive and inflammatory pain between male and female mice and quantify antinociception or analgesic effects and hyperlocomotive activities under morphine analgesia. The results demonstrate that the new TGT device and assay are sensitive and efficient for detecting nociception and pain in mice. The quality of data resulting from objective data collection and the rapid turnover time make this automated behavioral testing an attractive choice for preclinical pain investigations, particularly for *in vivo* screening of new analgesics. Additionally, the low cost of the TGT device makes it affordable for most laboratories and can generate a broad impact to the pain research field. Moreover, this enabling technology will help resolve current controversies surrounding the roles of various thermal sensing receptors in heat- and cold-induced pain, thereby accelerating *in vivo* pain evaluation and screening of new analgesics intended to target these receptors.

## Data Availability

The original contributions presented in the study are included in the article/Supplementary material, further inquiries can be directed to the corresponding author.
