# Supplementary material for: Automated thermal gradient test for unprovoked assessment of nociceptive preference in rodents
Source: Front Behav Neurosci. 2025 Dec 5;19:1709160. doi: 10.3389/fnbeh.2025.1709160 (PMC12714899; doi:10.3389/fnbeh.2025.1709160)
Supplement: Supplementary file 1 [file Data_Sheet_1.pdf]

Supplementary Materials

Automated thermal gradient test for unprovoked assessment of nociceptive preference in rodents

Thomas Deakin[1], Shoupeng Wei[1], Yao Wang[1], Raina E. Rhoades[1], Tommy S. Tillman[1], Pei Tang[1,2], Yan Xu[1,2,3,4*]

[1]Department of Anesthesiology and Perioperative Medicine, University of Pittsburgh, Pittsburgh, PA 15260, USA

[2]Department of Pharmacology and Chemical Biology, University of Pittsburgh, Pittsburgh, PA 15260, USA

[3]Department of Structural Biology, University of Pittsburgh, Pittsburgh, PA 15260, USA

[4]Department of Physics and Astronomy, University of Pittsburgh, Pittsburgh, PA 15260, USA

Short Running Title: Self-reported pain behaviors in mice

This file contains four supplemental figures and one supplemental table.

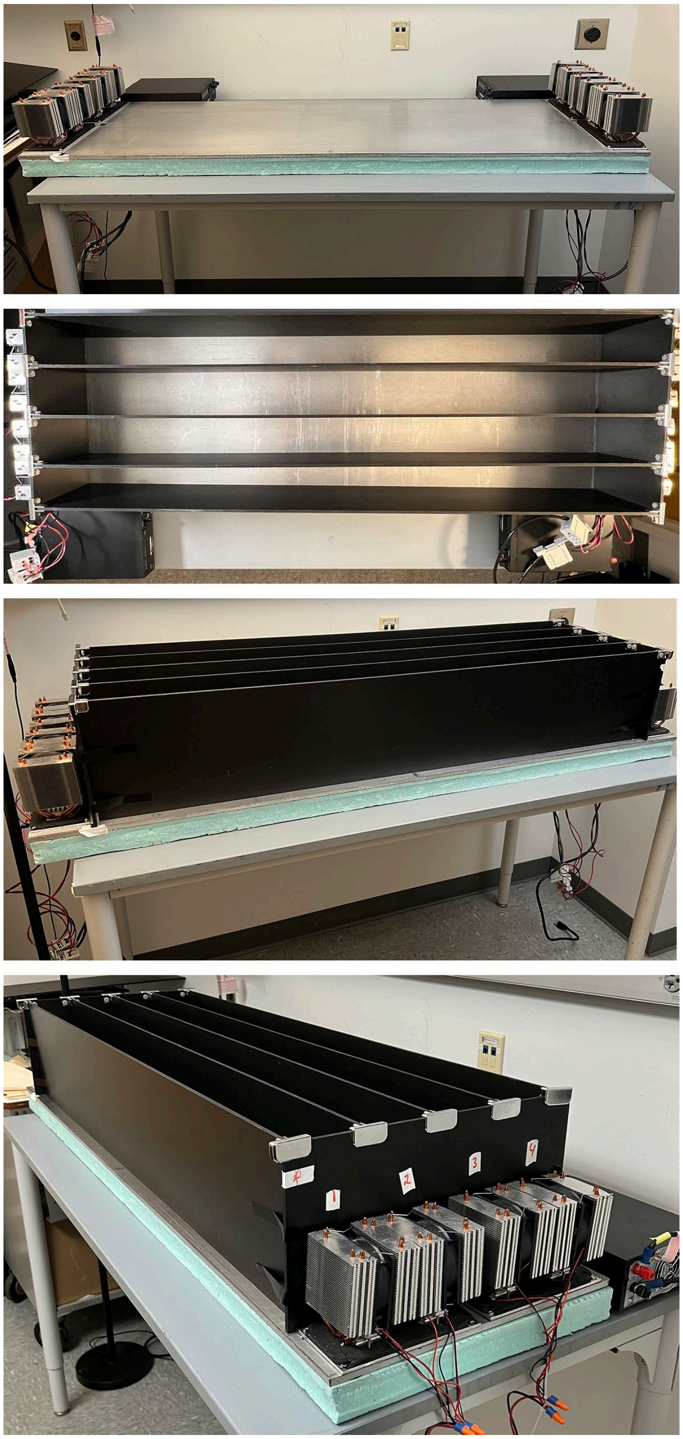

Supplementary Figure S1. Photos from different directions of the TGT device.

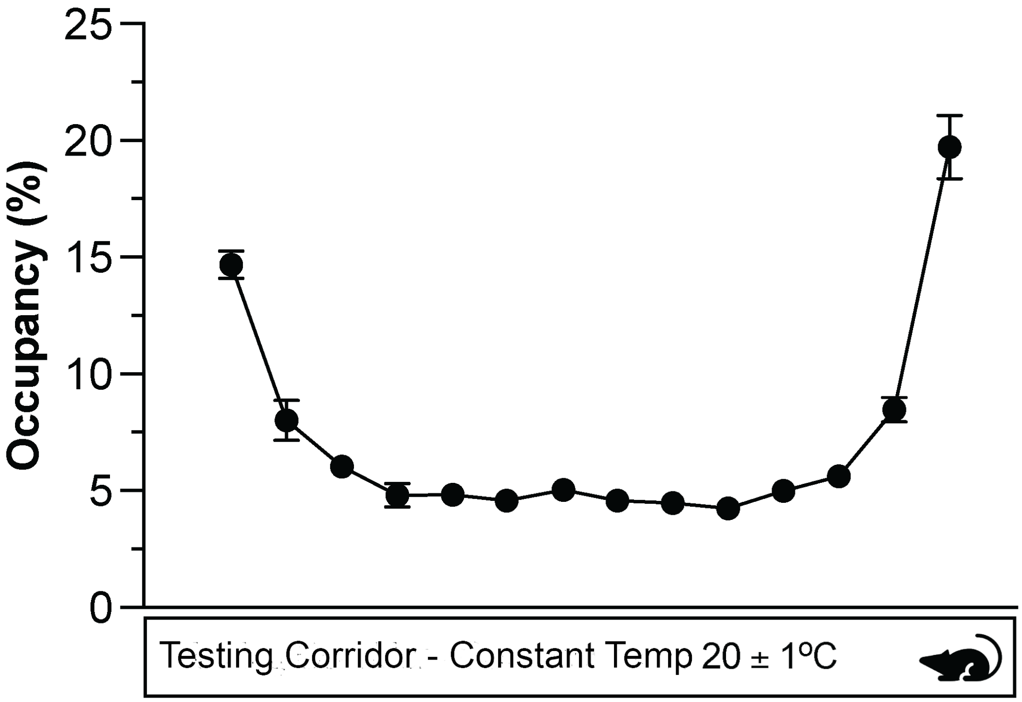

Supplementary Figure S2. **Habituation of mice in the TGT device at room temperature.** Habituation (10 min) of female C57BL/6J mice (n=29) inside the TGT device at 20 ± 1℃. The mice show a strong preference for the two ends of the long corridors so that the occupancy along the corridor exhibits a U shape; this is distinctly different from the bell-shaped Gaussian distributions shown in Figures 2-5 with an active temperature gradient. Male mice showed similar preference for corners at two ends.

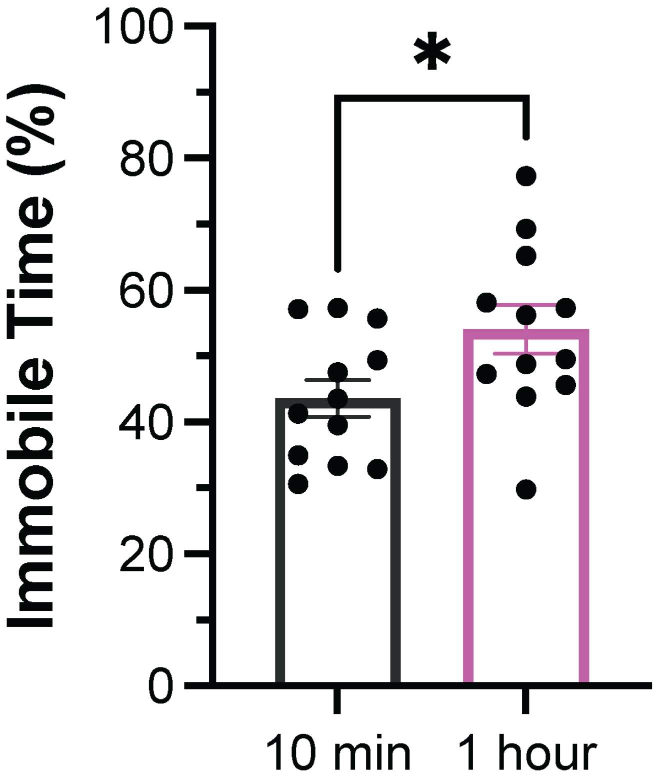

Supplementary Figure S3. **Comparison of accumulative resting time between 10 and 60 min TGT data acquisition.** The resting time, measured by the immobile time greater than 3 s, increased significantly during 1-h data collection as mice learned comfortable or preferred temperature zones and displayed a larger percentage of accumulative resting time during later data acquisition period. Data are Mean ± SEM (n = 12 from the same male mice as in **Fig. 2**).

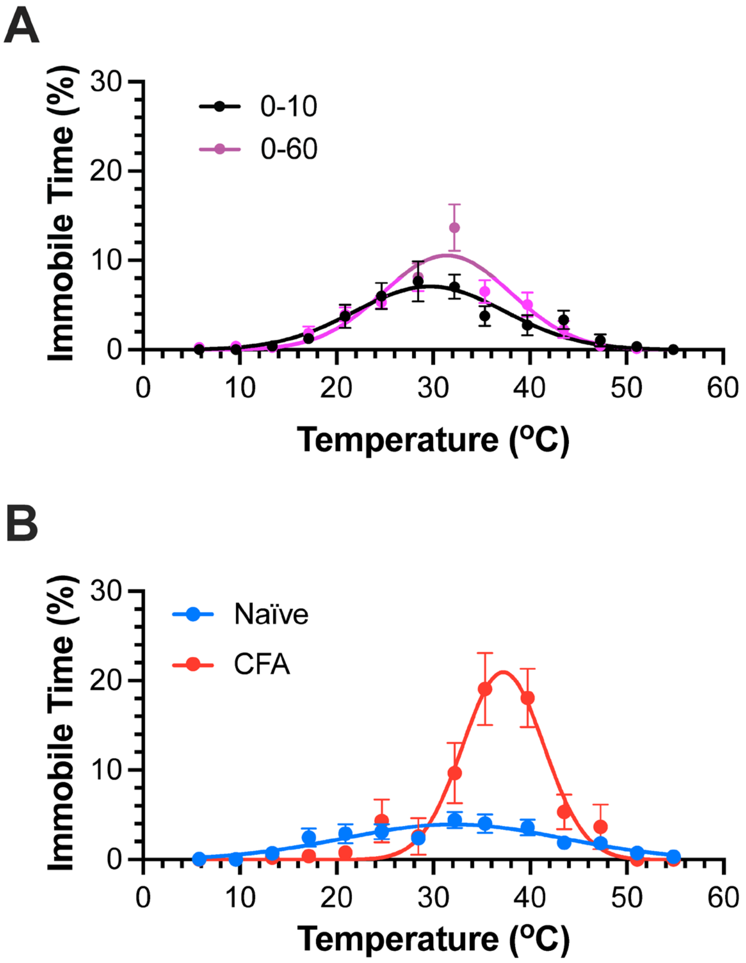

Supplementary Figure S4. **Distribution of >3 s immobilization (resting) of mice as a function of different temperature zones. (A)** While the animals quickly learned to be stationary in the preferred temperature zones (*i.e.*, remaining immobile for >3 s) during the first 10 min of data acquisition (black), this preference was reinforced as the acquisition time increased to 60 min (pink). Data are derived from the same n = 12 male mice as in **Fig. 2**, showing that the percentage of occupancy in the preferred zones is predominantly determined by the immobile times in these zones. (**B**) The zone distribution of immobilization >3 s for naïve female mice and for those with CFA-induced pain (n = 11 for each group) follows a similar pattern to the percentage occupancy in different temperature zones as shown in **Fig. 4**. The Gaussian fit shows significant difference between naïve and CFA mice ($p < 0.001$ from three-parameter nonlinear regression). Data are presented as Mean ± SEM.

Supplementary Table 1. A comparison between TGT and traditional assays

Feature	Thermal Gradient Test (TGT)	Hargreaves Test	Hot Plate Test
**Behavioral Naturalism**	High – allows free movement and choice	Low – stimulus is externally applied, and animal is confined with high anxiety	Low – animal is confined with high anxiety
**Throughput**	High	Low	Moderate
**Cost**	Low	High	Low
**Evaluator's Skill Required**	Low	High	Moderate
**Sensitivity to Locomotor Changes**	High – but movement data are useful	High – hyperactivity makes data unreliable.	Moderate – focused on reflex latency
**Space Required**	Moderate	Moderate	Small
**Animal Adaptation Time**	Short	Long	Short
**Data Analysis Complexity**	Moderate	Low	Low
